# Numerical Simulations of Components Produced by Fused Deposition 3D Printing

**DOI:** 10.3390/ma14164625

**Published:** 2021-08-17

**Authors:** Martina Scapin, Lorenzo Peroni

**Affiliations:** Department of Mechanical and Aerospace Engineering, Politecnico di Torino, Corso Duca degli Abruzzi, 24, 10129 Turin, Italy; lorenzo.peroni@polito.it

**Keywords:** fused deposition modeling, nylon reinforced with short fibers, tensile and bending experimental tests, transversely isotropic material behavior, finite element analyses for structural calculations

## Abstract

Three-dimensional printing technology using fused deposition modeling processes is becoming more and more widespread thanks to the improvements in the mechanical properties of materials with the addition of short fibers into the polymeric filaments. The final mechanical properties of the printed components depend, not only on the properties of the filament, but also on several printing parameters. The main purpose of this study was the development of a tool for designers to predict the real mechanical properties of printed components by performing finite element analyses. Two different materials (nylon reinforced with glass or carbon fibers) were investigated. The experimental identification of the elastic material model parameters was performed by testing printed fully filled dog bone specimens in two different directions. The obtained parameters were used in numerical analyses to predict the mechanical response of simple structures. Blocks of 20 mm × 20 mm × 160 mm were printed in four different percentages of a triangular infill pattern. Experimental and numerical four-point bending tests were performed, and the results were compared in terms of load versus curvature. The analysis of the results demonstrated that the purely elastic transversely isotropic material model is adequate for predicting behavior, at least before nonlinearities occur.

## 1. Introduction

With the advent of materials with increasingly high mechanical properties, three-dimensional (3D) printing by using fused deposition modeling (FDM) technique is evolving from purely aesthetic and/or functional prototypes to structural components. As a matter of fact, nowadays, it is possible to create components with mechanical properties such that they can be used under high loads for single prototypes or small series. This has been possible thanks to the continuous development of polymer composites, in which short fibers or nanomaterials have been added as reinforcement into the polymeric filament [1,2,3,4,5,6,7,8,9].

To improve the performance of the plastic composites and to extend their applications, continuous fiber-reinforced composite could be another option [8,9,10,11]. The first continuous fibers printer was developed by MarkForged© and the technology is in continuous development and improvement. Nowadays, several research groups print continuous fiber materials with their own printing heads. This technique requires printers much more expensive than the common ones. For this reason, the present work was focused on a more widespread solution which could be accessible to a greater number of users. The solution in which the filament produced by mixing short fibers with thermoplastic matrix (extruded together during printing) could be used with any printers in the market with few modifications (e.g., a nozzle with high wear-resistance to abrasive materials).

Usually, 3D printed components are not massive, but to save time and material (i.e., cost), the structures are made with an interior cellular structure. The density and pattern of the infill must be defined as printing parameters. The adoption of reticular infill structures has other advantages as well; it allows for maximizing the stiffness to weight ratio and considerably reduces the distortions of components that are induced during printing and subsequent cooling. Nevertheless, while such an approach can be easily understood, it has a weakness in that it does not provide the designer with direct control over the mechanical resistance of the component to be produced. This is because the morphology of the internal structure, the thickness of the walls, and other characteristics inevitably have a strong effect on the properties of the produced component. Most of these parameters are chosen during the printing preparation phase by slicing software [12,13] and are directly managed by the printer, which produces the machine language needed to correctly move the nozzle. As a result, the designer does not actually have the necessary tools to predict the behavior of the final components before printing. Often, the validation phase is performed directly with field tests on printed components. The problem is further complicated by the dependency of the final strength of the printed components on numerous other printing parameters and their combinations. Among them, the most influential of these parameters are the printing speed, the temperature and diameter of the nozzle, the base plate temperature, the height of the layers, the infill orientation, and of course, the build orientation. Many studies (e.g., [14,15,16,17,18]) have been carried out with the aim of demonstrating and analyzing the effects of one or more of these parameters on the resulting mechanical properties.

In the scientific literature, a lot of studies can be found aimed to the mechanical characterization of materials and printed parts, in case of composites. In [8,9,10,11,19], exhaustive reviews were presented in which the summary of the mechanical properties obtained for different materials, printing conditions and technologies were condensed and compared. Starting from the simple tensile test many authors extended the analysis changing the applied load (compression, bending, shear and torsion) and the loading history (fatigue, impact and creep). Depending on the material systems used, a wide variability of results can be found. For this reason, despite of the big amount of available data in the scientific literature, a lot of researchers preferred to perform the mechanical characterization directly taking into account the adopted material printing solution (e.g., material, printing parameters, technology, etc.).

**Figure 1:**
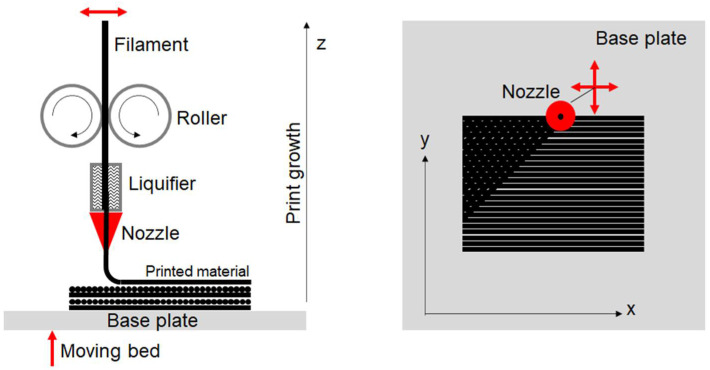
FDM printing scheme: The filament is extruded through the heated nozzle, which moves in the *x*-*y* plane, while the hot base plate moves in the z direction.

In general, although the filament is initially isotropic and homogeneous, it can be assumed that the layers printed with FDM behave as an orthotropic material. Some researcher applied the classical lamina/laminate theory to describe the behavior of a printed component [20,21,22,23,24,25,26]. In bulk printed components, each printing layer was obtained with aligned filament deposition. By stacking layers without changing the raster angle, the laminate structure could be considered a transversally isotropic material, in which the strong direction is that of the deposition, and the mechanical properties in the transverse planes are nearly isotropic. Conversely, by changing the raster angle between the stacked layers (Figure 1), the properties in the printing plane become more isotropic while weaker properties are observed in the growth direction, as reported in Table 1 and presented in [26]. In the elastic regime, the last behavior could be treated with a linear transversely isotropic model defined with a global reference system for the whole piece. According to the notation used in Figure 1, the *x*-*y* plane is the transverse isotropic plane and *z* is the direction of growth. However, when polymeric materials are used, it is expected that the mechanical behavior could immediately be strongly nonlinear and then assume the connotation of plastic (or rather, irreversible) anisotropic behavior [27]. Plasticity is not related to the motion of the dislocations, but to the evolution of the arrangement of the polymeric chains.

The last, but not least, aspect to be considered is that with this printing technique, it is not possible to eliminate intrinsic porosity due to the presence of voids between adjacent layers. The presence of voids depends on several parameters and in turn strongly affects the mechanical strength of the printed components. This also happens in fully filled portions, in which the component is obtained by stratifying the layers: the deposition of the (molten) filament produces a texture like that which is typical for a fabric [28,29,30,31].

Finite element (FE) method is a widely used tool for the analysis of structures produced by FDM. A great number of researchers modelled the process of deposition to evaluate cooling distortion and residual stresses induced (e.g., [32]). This approach is justified by the interest in obtaining the desired shape of prototypes, especially when the prototypes are not subjected to structural loads. In cases in which the components produced by FDM are place into operational conditions, the interest moved to the use of FE models to study the structural behavior. In some cases, the analysis regarded printed bulk components for which the material behavior was usually homogenized and the component was modeled with solid elements (e.g., [20,25,33]). An improvement of this approach could be the modelling of the bulk structure by reproducing each layer with the real deposition path (e.g., [34]): this produces models with an enormous number of elements, which could be solved only in case of small components. The two approaches could be combined, as in [23,25]: in this case the FE model of the whole component had material model parameters extracted from detailed models of the actual mesostructure. A great number of numerical studies were oriented to the analysis of lattice structures behavior (e.g., [35]) or to the use of topological optimization to find the best shape for the components or for the infill design (e.g., [36]).

The present study aimed to develop a technique that allows finite element modeling of structures produced by FDM by considering the real infill structure made during the printing phase. The goal was to create computationally efficient models for the structural/mechanical evaluation of components before production or putting into operation. The final aspect of the components made with this technique is that they have the typical structural connotation of thin-walled structures, even if with a more complex geometry. This consideration was the basis for the choice to build FE models by using shell elements for which the through thickness stress was not computed due to the plane stress state. In fact, the typical thickness of the infill structure is on the same order of magnitude as the diameter of the nozzle, which is between 0.2 and 0.8 mm.

## 2. Materials and Methods

The present study was dedicated to the investigation of the mechanical properties of components made with two different filaments: Nylforce Glass (NG) and Nylforce Carbon (NC), both produced by FiberForce (Treviso, Italy). Each has a nylon matrix that is reinforced by short fibers, making the materials extremely attractive because of the resultant combination of properties: noticeable tensile strength and stiffness, high levels of impact strength, good thermal stability, and high levels of resistance to chemical agents. In the first material, the filament is composed of nylon charged with glass fibers; in the second, the nylon matrix is reinforced with a high content of carbon fibers (approximately 20% by weight). A summary of the datasheet properties as declared by the manufacturer is reported in Table 1. In the following, *x* and *y* are the two directions on the print plane, and *z* is the growth direction, as shown in Figure 1.

**Figure 2:**
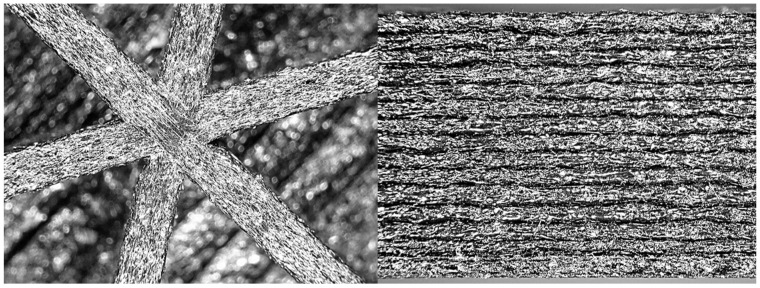
Optical microscope images of 3D structures printed with fiber-reinforced nylon. On the left, a view in the printing plane *x*-*y* of 1 mm thick walls. On the right, the side view of the layers in the *z* direction with a height of 0.25 mm.

In Figure 2, two different optical microscope images of 3D printed structures are provided. On the left side of Figure 2 there is the *x*-*y* view. It can clearly be seen that the orientation of the short fibers is in the direction of the extrusion. The right side of Figure 2 shows the *x-z* view, where the single layer has a height of 0.25 mm. Since the extrusion has a sort of rectangular/elliptical form [23,24,29,30,31], a void area remains between a layer and its neighboring layers, producing the typical lines visible on the printed surface (in both the *x*-*y* plane and *z* direction), and there is an apparent density reduction of the material. This is one of the reasons the printed structures exhibit less strength than expected, even at 100% infill. Because the printed structure is intrinsically porous, the measured mechanical properties (on a 100% infill specimen) are evaluated on the measured cross-section, hence they are averaged. The area reduction, like that observed in Figure 2, is globally perceived as a stiffness reduction, and consequently, there is a perceived reduction in elastic properties, as well as an underestimation of mechanical strength.

**Figure 3:**
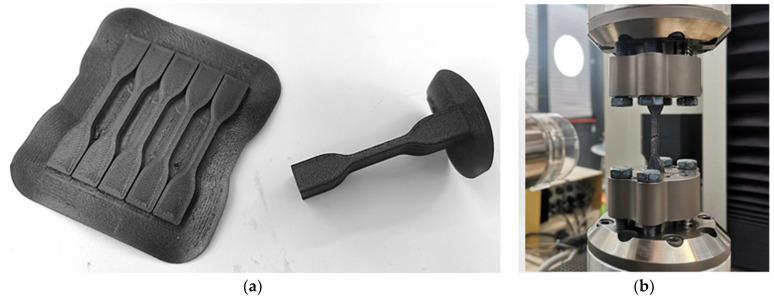
Tensile specimens printed with an Ultimaker S5 at 100% infill, with thickness in the *z* and *x* directions (**a**); image of a sample mounted in the testing machine (**b**).

To determine the mechanical properties to be used in the FE simulation for the prediction of the mechanical behavior of printed structures, in the present study, the authors performed tensile tests (Standard ASTM-E8M) on flat dog bone specimens (gage section of 4 mm × 4 mm and gage length of 25 mm) printed on an Ultimaker S5 with a print speed of 40 mm/s, an infill percentage of 100%, and an infill orientation of ±45°. Two series of specimens were printed (see Figure 3). The first batch (specimen P) had the thickness in the *z* direction, and the second batch (specimen Z) had the longitudinal axis in the *z* direction. The tests were performed on the standard testing machine Zwick Z05 (see Figure 3) at a speed of 0.5 mm/min. Each test was recorded by means of a high-resolution camera (2591 × 1944 pixels) at a frame rate of 2 fps.

The sequence of the images taken during each test was digitally elaborated to obtain the 2D state of deformation. Table 2 shows the ultimate strength values and the elastic constants obtained for the P and Z specimens. The same properties were obtained for the specimen with the longitudinal axis oriented in the *x* and *y* directions (P specimens) because the *x*-*y* plane is the plane of symmetry, while different values were obtained for the Z specimens. As expected, the P specimens showed greater strength and stiffness than the Z specimens. For the P specimens, the obtained mechanical strength was similar to the value obtained by performing tensile tests directly on the filament (these tests were performed on the same testing equipment used for the dog bone specimens). It is possible to conclude that the printing process slightly changed the material properties in the *x* and *y* directions (the difference could be mainly related to the induced porosity). Conversely, printing in the *z* direction significantly modified material properties. The Z specimens showed less strength and stiffness, mainly governed by the bonding between the layers deposited in the *z* direction, which is not as strong as bonding in the direction of the filament extrusion.

**Figure 4:**
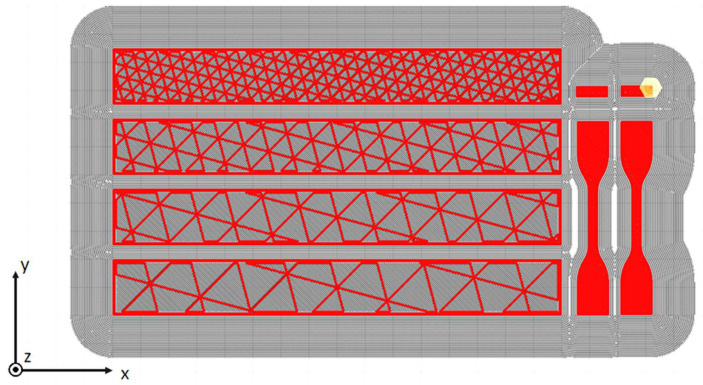
The slicing view of the specimens in Ultimaker Cura software (Version 4.9). The infill structure is made with a single material line and the external walls with two lines. The specimens for tensile tests were 100% filled with a ±45° alternate crossing pattern.

**Figure 5:**
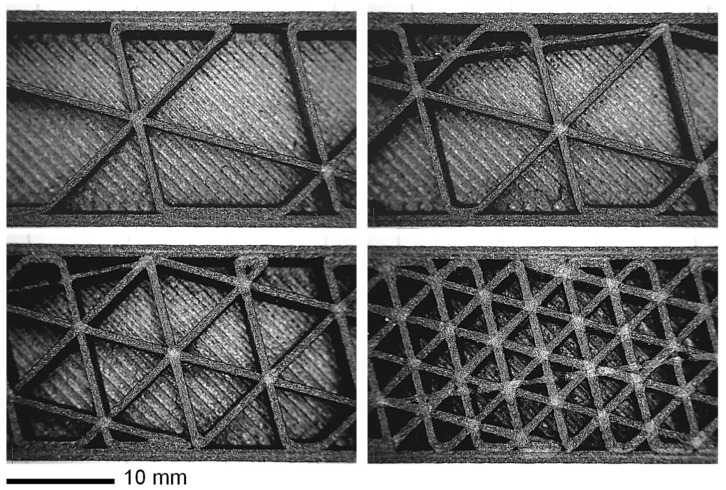
Optical microscope images of the four types of infill for bending samples: 16, 12, 8, and 4 mm of distance between the parallel lines of the pattern.

The main goal of the actual work was to reproduce in FE simulations the mechanical properties expected for structures printed by additive manufacturing. To demonstrate the feasibility of the technique, simple structures were printed (see Figure 4), specifically, 20 mm × 20 mm × 160 mm blocks for four-point bending tests. The dimensions of the benchmark structure were chosen to be representative of components printable with common desktop printers (with a printing volume of in the order of 200 mm × 200 mm × 200 mm). A bending loading condition was adopted to create a non-uniform stress distribution inside the material reproducing in the laboratory what could happen in a printed component subjected to operational structural loads. In addition, the bending test also allowed the evaluation of the mechanical stiffness in a more complex condition than a simple tensile test being also more sensitive to the infill. Moreover, the dimensions and the loading configuration were chosen to minimize the local effects produced by the rollers (indentations): the components are substantially complex thin-walled structures (Figure 5) and could be easily subjected to local buckling effects. Some preliminary tests with different configurations were performed to define the final adopted setup.

The specimens were printed with four different infill percentages using a triangular pattern (see Figure 4). During the printing model preparation, the distance between parallel lines (i.e., the height of the equilateral triangles) of the infill was imposed (4, 8, 12, and 16 mm), from which the infill density was derived. The imposed printing parameters are reported in Table 3. As shown in Figure 4, each batch of printing was composed by four bending specimens, two P specimens, and two Z specimens (the P and Z specimens were printed at 100% of infill, as previously mentioned).

The images of the infill structures were obtained with an optical microscope (see Figure 5). These images were used to precisely measure the real wall thickness (measured thickness) obtained from the printing. The measured values are reported in Table 4 in comparison with the imposed values (nominal thickness). The measured thickness differed substantially from the imposed thickness. The images in Figure 5 clearly show the paths followed by the nozzle during the deposition and repositioning phases.

For each bending specimen, the mass was also measured. The average values from the different batches are reported in Table 5 for both the materials and the four infill densities.

**Figure 6:**
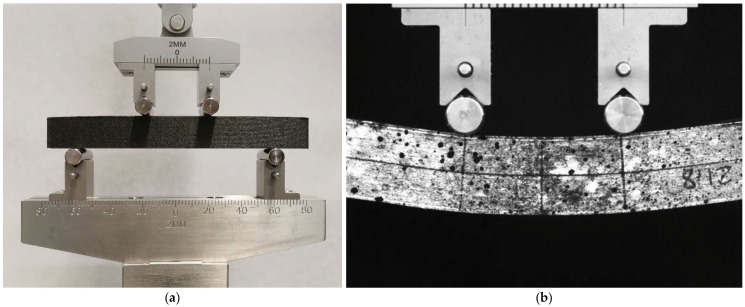
Experimental setup for four-point bending tests on 3D printed structures (**a**). For each sample, the image sequence of the test was acquired with a 5 MP Pixelink USB camera, and the surface of the sample was painted to allow digital image processing and obtain the displacements (**b**).

The experimental bending tests were performed on an electromechanical testing machine Zwick Z05 (Zwick Gmbh & Co, Ulm, Germany). The experimental setup was reported in Figure 6. The tests were performed by imposing a speed of 5 mm/min on the support of the upper rollers. Each test was recorded by means of a Pixelink camera with a 5 MPixels of resolution at a frame rate of 2 fps. A speckle was produced on the lateral surface of each specimen to allow for digital image processing of the videos, and a software for tracking was used to obtain the effective displacements imposed on the specimens. The displacements of the lower part of the specimen were fitted with a circle to get the curvature time history profile induced by bending. As described in Section 3, the diagrams for the experimental force versus the curvature were used for the comparison with the numerical results.

**Figure 7:**
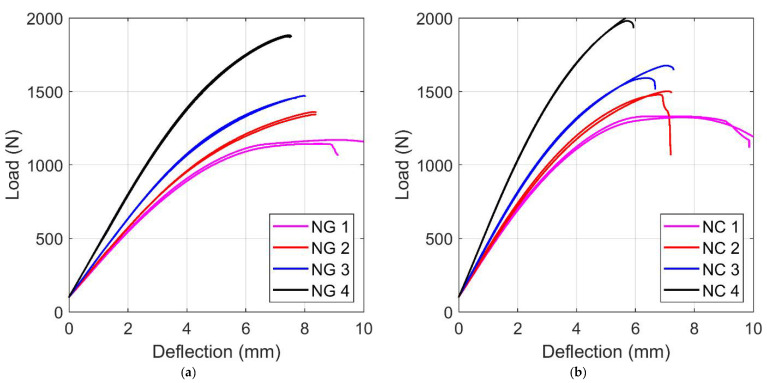
Results of the experimental bending tests for the two materials: (**a**) Nylforce Glass (NG) and (**b**) Nylforce Carbon (NC). For each configuration, the results of the two samples are reported to show good repeatability under all testing conditions.

A series of repetitions were performed on the NG and NC specimens. Figure 7 shows the results of two tests for each infill percentage reported in terms of force versus deflection directly obtained by the testing machine. There was very good repeatability of the results for all the testing conditions. As expected, by reducing the infill density the strength decreases and the deflection increases. In addition, the analysis of the results reported in Figure 7 shows that the material behavior was, as expected, highly nonlinear, with a plastic softening response. Nevertheless, in the first part of the test, the behavior could be described with a linear elastic model with a good level of accuracy.

### 2.1. Numerical Modeling of Printed Structures

To correctly model the structures obtained with the FDM process using commercially available printers, the geometries of both the structure and the infill need to be known. A few slicing software directly export the geometry of the infill. Prusa, an open-source application, can directly export the STL file for each part of the printed model that contains geometric information. Other applications, for example Cura, do not give this possibility, but only allow for exporting the G-code with the tool path data, which must then be processed to acquire the desired information about the printed infill.

Independently from the choice of the slicing software, it is necessary to understand how to convert the geometry model into the FE model (nodes and elements). Typically, an STL geometry file requires a series of operations for simplification and/or smoothing/polishing because this format is typically optimized for visualization purposes rather than calculation. In addition, it is necessary to consider that the printed object is a 3D structure, while the triangularization mesh is usually defined on the external surface.

From the point of view of the structural/mechanical calculation, a printed structure can be modeled with both solid and shell elements. In the first case, the risk is obtaining too many elements to correctly appreciate the bending behavior. In the second case, the membranal and flexural behaviors could be evaluated with a more limited number of elements, but the risk is not correctly modeling the real geometry, the presence of intersections between walls, and so on.

The numerical simulations were carried out using LS-DYNA code, and nonlinear static implicit analyses were performed. In the present work, the authors chose to adopt the shell elements because the printed structures were characterized by low thickness (in the order of magnitude of 1 mm). A shell element with a fully integrated formulation was used.

**Figure 8:**
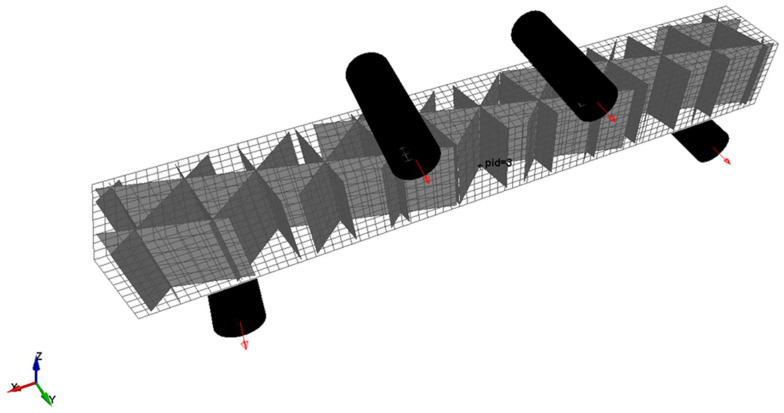
Finite element (FE) 3D model of the bending test (infill ID 1).

**Figure 9:**
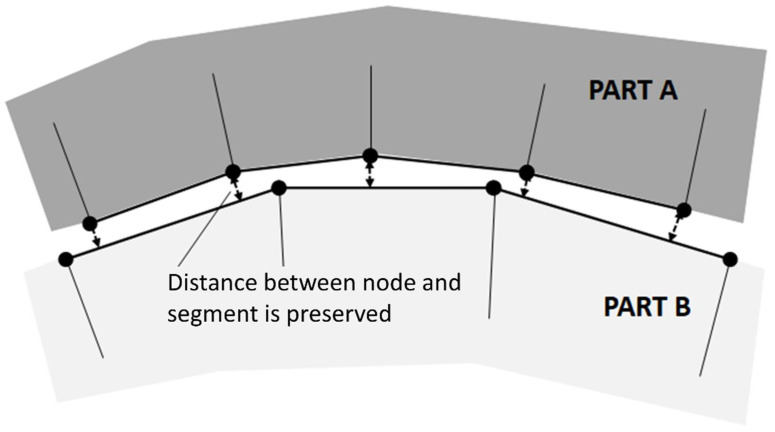
The tied contact algorithm.

The entire model was composed of two parts: the external surface obtained from the CAD model of the component and the infill obtained by extruding the slicing pattern. The latter was generated in accordance with its exact orientation and position as in the *x*-*y* plane of the printer. Then, a Boolean subtraction operation was applied to remove the surplus of infill. After this operation, the two parts were independently meshed. For the specimens used in the present work (Figure 8), this phase produced a regular mesh in both parts, but this operation typically produces regular mesh for the infill, while free mesh algorithms could be necessary for the external surface, depending on its complexity. The two parts were joined by means of a tied contact, able to couple displacement and rotation by also considering the gap between the parts (Figure 9 [37]). With this method, the nodal connectivity is not required; hence, the mesh density and nodal position can be different in the two parts. In particular, the contact was defined between the external nodes of the infill and the shell plane of the external surface.

The entire model also included the rollers that were in contact with the entire structure and upon which the boundary conditions were imposed. A prescribed motion was imposed on the two upper rollers in terms of vertical displacement, while the lower rollers were fixed (Figure 8).

### 2.2. Material Modeling

A fundamental aspect in the numerical modeling of 3D printed structures is represented by the material modeling. Several approaches have been proposed [38] for correctly modeling the mechanical behavior of FDM printed materials. As discussed in Section 2, the materials investigated in the present study exhibited different properties in the two main directions that were tested. As previously anticipated, in the case of FDM short fiber-reinforced printed components, the growth direction (in this case, the *z* direction) has fewer mechanical properties. This is for many reasons, which can be summarized as follows:

1. The bond between the layers is weaker than the bond in the direction of extrusion. The hot fused material is deposited on the previously deposited colder layer, which reduces the strength of the bond between the two portions.
2. The reinforced fibers are primarily oriented in the direction of the extrusion (Figure 1 and Figure 2).
3. The obtained structure is not regular and presents a section reduction in the connection between the two layers (Figure 1 and Figure 2).

In this work, this behavior was described by using a transversely isotropic model. In the body of scientific literature, this material model is widely used for describing the mechanical response of a special class of orthotropic materials, which, as in the present case, have isotropic properties in one plane (in which the elastic properties will be defined herein as the in-plane) and different properties in the normal direction of this plane (in which the elastic properties will be defined herein as transverse).

The constitutive model for an elastic transversely isotropic material can be derived from the generic elastic orthotropic equation expressed by Equation (1):

(1) $$\left\{\begin{array}{c} \varepsilon_{xx}\\ \varepsilon_{yy}\\ \varepsilon_{zz}\\ \gamma_{xy}\\ \gamma_{yz}\\ \gamma_{zx} \end{array}\right\}=\left[\begin{array}{c} 1/E_{xx}\\ -\nu_{xy}/E_{xx}\\ -\nu_{xz}/E_{xx}\\ 0\\ 0\\ 0 \end{array}\begin{array}{c} -\nu_{yx}/E_{yy}\\ 1/E_{yy}\\ -\nu_{yz}/E_{yy}\\ 0\\ 0\\ 0 \end{array}\begin{array}{c} -\nu_{zx}/E_{zz}\\ -\nu_{zy}/E_{zz}\\ 1/E_{zz}\\ 0\\ 0\\ 0 \end{array}\begin{array}{c} 0\\ 0\\ 0\\ 1/G_{xy}\\ 0\\ 0 \end{array}\begin{array}{c} 0\\ 0\\ 0\\ 0\\ 1/G_{yz}\\ 0 \end{array}\begin{array}{c} 0\\ 0\\ 0\\ 0\\ 0\\ 1/G_{zx} \end{array}\right]\{\begin{array}{c} \sigma_{xx}\\ \sigma_{yy}\\ \sigma_{zz}\\ \tau_{xy}\\ \tau_{yz}\\ \tau_{zx} \end{array}\}$$

where the symmetry condition of the compliance/stiffness matrices necessitates

(2) $$\frac{\nu_{ij}}{E_{ii}}=\frac{\nu_{ji}}{E_{jj}}$$

The elastic transversely isotropic model is completely described by five independent elastic constants rather than the nine to be determined in the case of orthotropic models. The five independent parameters that need to be experimentally evaluated are the in-plane elastic modulus $E_{xx}=E_{yy}$, the in-plane Poisson’s ratio $\nu_{xy}=\nu_{yx}$, the transverse elastic modulus $E_{zz}$, the transverse Poisson’s ratio $\nu_{zx}=\nu_{zy}$, and the transverse shear modulus $G_{zx}=G_{yz}$. The in-plane shear modulus can be obtained starting from the in-plane elastic modulus and Poisson’s ratio as $G_{xy}=\frac{E_{xx}}{2\left(1+\nu_{xy}\right)}$.

The elastic transversely isotropic behavior was modeled with *MAT_002 defined in LS-DYNA [37]. During the numerical solution, the stress update is performed in the local coordinate system of the element. In case of shell elements associated with an anisotropic material, it is necessary to define the initial direction of the material axes which will be updated in accordance with the element rotation and deformation. In LS-DYNA, the material axes are identified by the *a-b-c* system, which, in case of shell elements, is defined as follows: *c* is the thickness direction, *a* is in the plane of the shell, and *b* = *c* × *a* (cross product). The definition of a generic orthotropic behavior using the material model *MAT_002 requires defining nine elastic properties, which in the coordinate system of the material are defined as: $E_{a}$, $E_{b}$, $E_{c}$, $\nu_{ba}$, $\nu_{ca}$, $\nu_{cb}$, $G_{ab}$, $G_{bc}$, and $G_{ca}$. In addition, it must be considered that because the shells are plane stress elements, the material parameters $E_{c}$, $\nu_{ca}$, and $\nu_{cb}$ are not used in the calculation.

**Figure 10:**
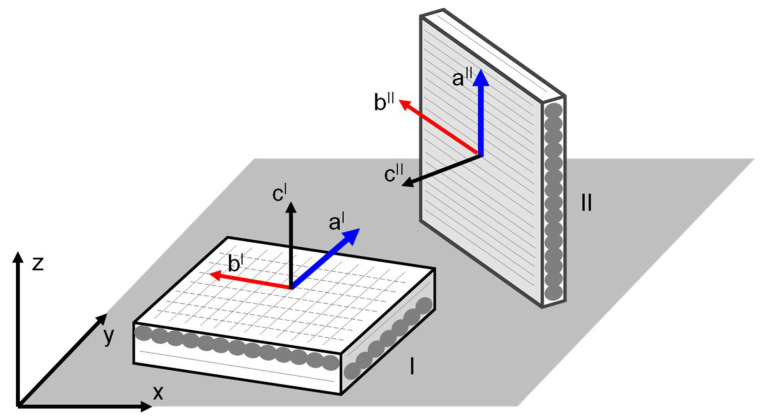
Material axes versus global axes definitions: case I, the plane of the shell is defined in the *x*-*y* global plane; case II, the plane of the shell is normal to the *x*-*y* global plane.

For the simple case presented in this paper, as can be seen in Figure 8, the shell elements in the undeformed geometry were oriented in two ways: Figure 10 is a schematic view of the two situations. The shell elements on the top and bottom surfaces of the specimen (which were in contact with the rollers) had the plane of the shell defined in the global *x*-*y* plane, while the thickness direction was aligned with the global *z* direction (referred to case I in Figure 10). The shell elements on the lateral surfaces of the specimen and those of the infill had an edge of shell elements that was parallel to the *z* direction and the plane of the shell was normal to the *x*-*y* global plane (referred to case II in Figure 10). For the situation shown as case I of Figure 10, $E_{a^{I}}=E_{b^{I}}=E_{xx}$ and, due to the plane stress hypothesis, the material became isotropic (i.e., no further parameters needed to be defined except for $\nu_{a^{I}b^{I}}=\nu_{b^{I}a^{I}}=\nu_{xy}$ and $G_{a^{I}b^{I}}=\frac{E_{a^{I}}}{2\left(1+\nu_{a^{I}b^{I}}\right)}$). For the situation shown as case II of Figure 10, $E_{a^{II}}=E_{zz}$ and it was assumed that $E_{b^{II}}=E_{xx}$ and $\nu_{b^{II}a^{II}}=\nu_{xz}$. The transverse shear modulus $G_{a^{II}b^{II}}\;$could be approximately evaluated starting from the other elastic constants. As reported in [39], for orthotropic plates, Huber proposed that the shear modulus could be evaluated starting from the in-plane elastic moduli and the Poisson’s ratios. For the shells of case II, this lead to

(3) $$G_{a^{II}b^{II}}=\frac{\sqrt{E_{a^{II}}E_{b^{II}}}}{2\left(1+\sqrt{\nu_{a^{II}b^{II}}\nu_{b^{II}a^{II}}}\right)}$$

which allowed to reduce the experimental efforts to simple tension tests in two printing configurations (longitudinal axis of the specimen in the *x*-*y* printing plane and longitudinal axis of the specimen in the *z* direction). The Huber approximation could be replaced by the direct determination of the shear modulus from pure shear tests (which in general are quite complex) or by the use other indirect solutions based on tensile tests performed on samples printed with different orientation, as performed in [26]. Table 6 reports the material model constants defined in the numerical models.

## 3. Results and Discussion

**Figure 11:**
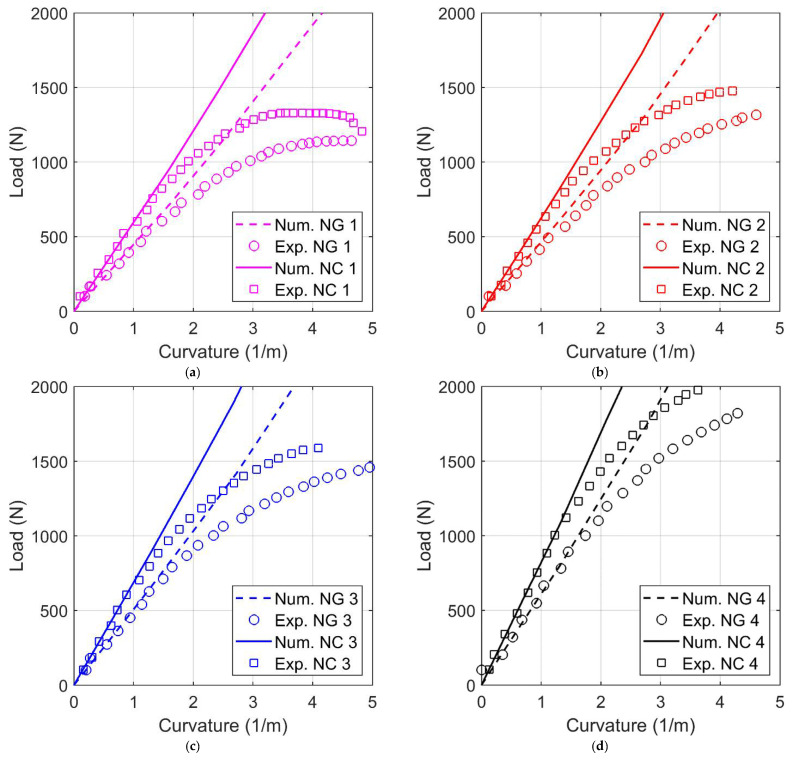
Comparison of experimental and numerical results in terms of load vs. curvature for the two investigated materials (Nylforce Glass (NG) and Nylforce Carbon (NC)) and for the four infill geometries (1 = 16 mm (**a**); 2 = 12 mm (**b**); 3 = 8 mm (**c**); 4 = 4 mm (**d**)).

Figure 11 provides a summary of the results obtained by the numerical simulations compared with the experimental ones. To remove the experimental results from spurious deformations due to the test equipment, a local deformation measurement was used instead of macroscopic and global quantities. The comparison between the experimental and numerical results was performed by measuring the time history of the curvature of the specimen. In particular, the curvature of the central portion of the specimen (i.e., between the central rollers) was investigated. The coordinates of three nodes placed on the bottom of the lateral surface with shells in the *z-x* plane were fitted with a circle. Then, a diagram was obtained of the load versus curvature (calculated as 1/*R*, where *R* is the radius of the fitting circle) by repeating this procedure for the entire deformation process. The same approach was used for both the experimental and numerical results.

The diagrams shown in Figure 11 reveal that the model was able to reproduce the behavior of the real components with a good level of accuracy for what concerns the global stiffness of the investigated materials (NG and NC) and for all the infill geometries. Because the material model used is purely elastic, it was not able to follow the experimental results once the plasticization of the sample became important.

**Figure 12:**
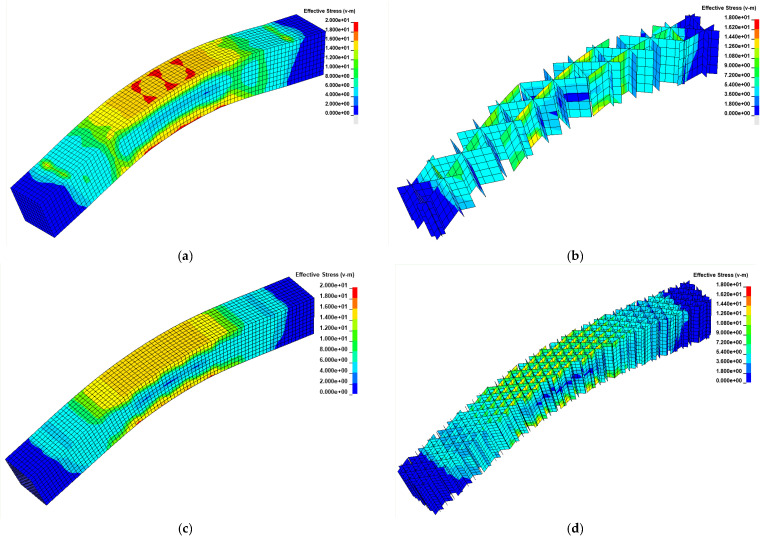
Spatial distribution of effective stress (MPa): (**a**) on the walls for NC2 specimen; (**b**) on the infill for NC2 specimen; (**c**) on the walls for NC4 specimen; (**d**) on the infill for NC4 specimen. The distributions correspond to an imposed displacement of 3 mm. The displacements in the figures were amplified by a factor of 5.

**Figure 13:**
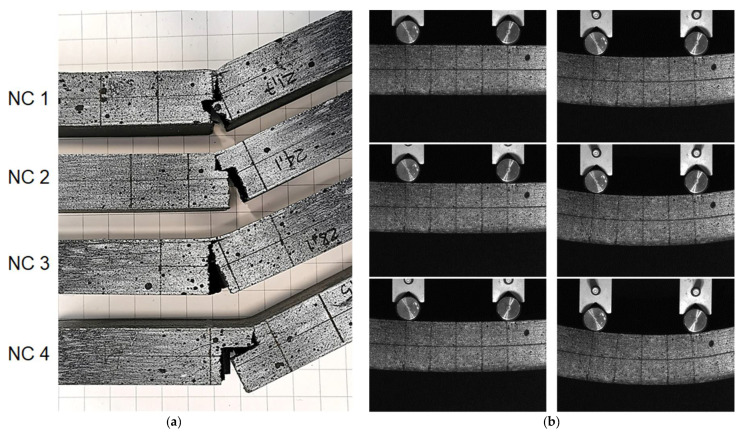
Images of the fracture obtained with a batch of NC specimens (**a**). Sequence of images (**b**) recorded during a test on the NC4 specimen (time interval between images is about 18 s).

In Figure 12, the effective (Von Mises) stress distribution is reported for two different geometries (infill ID 1 and 4) on the external surface and the infill. The numerical results are reported at the time corresponding to the linear behavior of the specimens, and the displacements were amplified by a factor of 5. As expected, in the central portion of the specimen, a linear variation of the axial stress was obtained (traction on the bottom surface and compression on the top surface). The situation is the same for all sections between the two central rollers due to the constant bending moment (shear load is null). The mechanical behavior of lateral surfaces and infill results from the combination of compression in the global *z* direction and bending stress, in accordance with the material properties. The right side of Figure 13 shows a sequence of images recorded during an experimental test performed on the NC4 specimen.

As expected, increasing the infill density caused an increase in the stiffness of the structure. Moreover, due to the distribution of the infill support on the top and bottom surfaces, the stress localization at the surface/infill interfaces was reduced. However, the increase in the component stiffness reduced the possibility of wall collapsing, decreasing global deformation at failure. Because the behavior of the material is quite ductile, the wall instability and crushing could be an efficient way of dissipating energy during component failure; a high-density infill reduces this phenomenon in the case of bending loads.

The failure of the components is always dominated by the tensile fracture of the bottom surface (see Figure 13a), which is oriented with the strong direction parallel to the induced stress. After the failure of the bottom surface, the crack propagated in the lateral walls (in direction *z*) and in the infill, with the possibility of some abrupt change of direction produced by interplane fracture. Interplanar failures were not observed before the propagation of the first crack in the sample. During the loading phase, the stress state in the infill and lateral surfaces in the *z* direction were substantially a compression and interplanar failure could not occur.

## 4. Conclusions

In the present work, a possible modeling technique for 3D printed parts using FDM technology was presented. The advent of several high-strength materials has caused widespread use of this manufacturing technique for components and structures and not only for prototypes. In most cases, the components are not printed with an infill percentage of 100%. A common solution is to use a reticular internal structure. This allows savings in materials, time, and other costs, but it also prevents, or at least limits, distortions.

The infill structures are usually obtained by extruding specific paths to reduce the complexity of the printing. The internal patterns are directly created by the slicing software, which generates the machine language (G-code) needed to control the movements of the nozzle.

Designers do not typically perform structural calculations for the components that will be printed in such a manner, nor do they optimize the printing parameters to maximize structural performance. Mainly, these steps are omitted due to difficulties related to the transformation of the 3D printing model into an adequate model for structural calculations and analysis.

In the present work, a simple structure, a block measuring 20 mm × 20 mm × 160 mm, was printed, and experimental four-point bending tests were performed. Two different materials (nylon reinforced with glass fibers, called Nylforce Glass (NG), and nylon reinforced with carbon fibers, called Nylforce Carbon (NC)) and four infill densities were investigated for a triangular infill pattern. The same components were also modeled for Finite Element Analysis using LS-DYNA code. The numerical models were obtained using shell elements, and the infill patterns were automatically built based on the elementary cell. Conversely, the external surface of the component was directly obtained from the STL file. The infill was then connected to the external surface of the component by defining a tied-type contact. This algorithm allows for a connection between meshes with different dimensions and node positions and is a widely used technique for modeling structural bonding. In addition, because the adopted approach makes the internal and external meshing phases independent, it also makes independent the variations between the two parts (e.g., it is easy to modify the infill pattern and its density).

As is well known, the component produced by FDM technology results in intrinsic anisotropic behavior. Under the assumption that the properties in the printing plane are isotropic and the mechanical properties in the transverse direction (i.e., the print growth direction) are weaker, a transversely isotropic material model was chosen as constitutive law. Although a nonlinear plastic behavior was observed during the tests, a purely elastic material model was adopted as a preliminary approach. This means that the material model was completely defined by five independent parameters. These parameters were obtained from tensile tests performed on sheet dog bone specimens printed contextually with the blocks for bending tests. In the same printing batch, specimens were printed with the thickness in the *z* direction and with the longitudinal axis in the *z* direction.

The experimental results obtained from bending tests were compared with the numerical results in terms of load versus curvature, which was obtained by fitting with a circle the zone where there were traction fibers in the central portion of specimen. The analyses of the results led to the conclusion that the numerical models were able to predict the linear phase of deformation with a good level of accuracy and were able to correctly estimate the stiffness of the components. In addition, they also allowed for evaluation of the stress distribution in the different parts and directions of the components.

## Figures and Tables

**Table 1:** Summary of the datasheet properties for Nylforce Glass (NG) and Nylforce Carbon (NC), as declared by the manufacturer, FiberForce.

Property (Units)	NG		NC	
	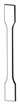 xz	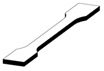 xy	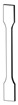 xz	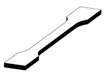 xy
Tensile strength (MPa)	n.a.	64.7	12.64	66.3
Elastic modulus (MPa)	n.a.	2534	1513	2758
Elongation at break (%)	n.a.	9.2	2.0	6.7
Energy at break (J)	n.a.	18.7	0.64	12.2
Density (g/cm^3^)	1.07		1.00	
Melting point (°C)	180		180	

Note: Tensile tests performed on specimens printed on Ultimaker 2+ with Olsson Ruby nozzle, nozzle temperature of 260 °C, head bed temperature of 70 °C, print speed of 40 mm/s, infill percentage of 100%, and infill orientation of 45°.

**Table 2:** Summary of material properties obtained from experimental tensile tests on the printed sample (100% infill) and filaments.

Material	$E_{xx}=E_{yy}\;(\mathrm{MPa})$	$E_{zz}$ (MPa)	$\nu_{xy,xz,yz}$(−)	${\sigma_{u,}}_{x}={\sigma_{u,}}_{y}\;(\mathrm{MPa})$	${\sigma_{u,}}_{z}$ (MPa)	${\sigma_{u,}}_{filament}\;(\mathrm{MPa})$
NG	1470	1174	0.4	41.8	23.2	52.2
NC	2215	1200	0.4	53.8	39.4	62.7

**Table 3:** Main print settings.

Printer	Ultimaker S5
Print core	CC Red 0.6 (Rubin)
Filament diameter	2.85 mm
Layer height	0.25 mm
Wall thickness	0.8 mm
Top/bottom thickness	0.8 mm
Infill line distance	16/12/8/4 mm
Infill layer thickness	0.25 mm
Printing temperature	270 °C
Build plate temperature	60 °C
Print speed	45 mm/s

**Table 4:** Nominal and measured thickness of printed samples.

Part	Nominal Thickness (mm)	Measured Thickness (mm)	
		NG	NC
Infill	0.25	0.9	0.85
Wall	0.8	1.4	1.2
Top	0.8	1	1
Bottom	0.8	1.4	1.2

**Table 5:** Measured masses of printed samples.

Infill ID	Line Distance (mm)	Mass (g)	
		NG	NC
1	16	21.95	21.75
2	12	24.35	24.18
3	8	28.30	28.15
4	4	40.85	40.55

**Table 6:** Material model parameters used in numerical simulations.

Material	$E_{a^{I}},E_{b^{I}},E_{b^{II}}\;(\mathrm{MPa})$	$\nu_{a^{I}b^{I}}\;$(−)	$G_{a^{I}b^{I}}\;(\mathrm{MPa})$	$E_{a^{II}}\;(\mathrm{MPa})$	$\nu_{b^{II}a^{II}}\;$(−)	$\nu_{a^{II}b^{II}}\;$(−)	$G_{a^{II}b^{II}}\;(\mathrm{MPa})$
NG	1470	0.4	525	1174	0.4	0.32	484
NC	2215	0.4	791	1200	0.4	0.22	630

## Data Availability

The raw/processed data required to reproduce these findings cannot be shared at this time as the data also forms part of an ongoing study.
